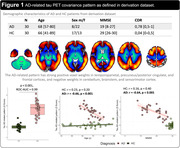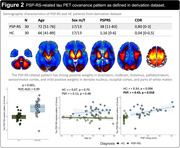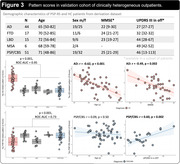# Voxel‐wise Florzolotau (APN‐1607) Tau PET Covariance Patterns in Alzheimer’s Disease and Progressive Supranuclear Palsy

**DOI:** 10.1002/alz.092585

**Published:** 2025-01-09

**Authors:** Ganna Blazhenets, Joachim Brumberg, Hironobu Endo, Jung‐Lung Hsu, Lars Frings, Nils Schroeter, Sabine Hellwig, Chiung‐Chih Chang, Makoto Higuchi, Philipp Tobias Meyer

**Affiliations:** ^1^ Medical Center ‐ University of Freiburg, Faculty of Medicine, University of Freiburg, Freiburg, Baden‐Wuerttemberg Germany; ^2^ National Institutes for Quantum Science and Technology, Chiba Japan; ^3^ Chang Gung Memorial Hospital Linkou Medical Center, Taoyuan Taiwan; ^4^ Cognition and Aging Center, Kaohsiung Chang Gung Memorial Hospital, Kaohsiung Taiwan

## Abstract

**Background:**

Florzolotau (APN‐1607) tau‐PET has shown distinct patterns of binding in patients with AD and 4‐repeat tauopathies. We aimed to establish disease‐specific tau covariance patterns in AD and PSP/CBS and validate them as user‐independent quantitative biomarkers for reference‐region‐free evaluation of tau‐PET in an independent clinical cohort.

**Method:**

We analyzed Florzolotau PET data from four different cohorts. The derivation dataset included 30 Aβ‐ healthy controls (HC) and 30 patients each with Aβ+ AD and PSP with Richardson’s syndrome (PSP‐RS). The validation dataset included 44, 51, 6, 15, and 17 patients with AD, PSP/CBS, MSA, LBD, and FTD, respectively. First, we applied scaled sub‐profile modelling principal components analysis to the derivation dataset to define the AD‐ and PSP‐RS‐related covariance patterns. Second, the topographic profile rating algorithm was applied to each scan to calculate AD‐ and PSP‐RS‐related pattern scores.

**Result:**

The AD‐ and PSP‐RS‐related patterns are described in Figures 1 and 2. In the derivation dataset, the AD‐ and PSP‐RS‐scores showed strong increases compared to HC (both p<0.001, AUC‐ROC=0.994 and 0.898, respectively). AD‐scores showed a significant negative association with age (r=‐0.66, p<0.001) and MMSE (r=‐0.64, p<0.001) in AD, while PSP‐RS‐scores correlated with the PSP rating scale (r=0.43, p=0.018) in PSP‐RS. In the validation dataset (Figure 3), AD‐scores were highest in AD (p<0.001 vs. all other, AUC‐ROC[AD vs. all other]=0.946) and were negatively associated with age and MMSE in AD (r=‐0.62, p<0.001 and r=‐0.49, p=0.002, respectively). PSP‐RS‐scores were highest in the PSP/CBS (p<0.001 vs. all other [at least p<0.05 vs. each group], AUC‐ROC[PSP/CBS vs. all other]=0.734), though also relatively increased in FTD and MSA. UPDRS‐III scores (off‐state) were available in 23 PSP/CBS patients and significantly associated with PSP‐RS‐scores (r=0.60, p=0.002).

**Conclusion:**

Modelling with principal components analysis allows for reference‐region‐free quantification of tau imaging data by constructing disease‐specific covariance patterns. When applied to Florzolotau, AD‐ and PSP‐RS‐related patterns showed high discrimination power in both the derivation and the clinically heterogeneous validation cohorts. PSP‐RS‐related pattern expression in FTD and MSA may be related to PSP pathology and known striatal off‐target binding, respectively. Strong association with respective disease severity scores further advocates for use of AD‐ and PSP‐RS‐related patterns as potential quantitative biomarkers of tau pathology.